# Immunohistochemical Expression of Laminin 332 in Triple-Negative Breast Carcinoma: A Cross-Sectional Study

**DOI:** 10.7759/cureus.92206

**Published:** 2025-09-13

**Authors:** Kamala Kannappalli, Kalyani Raju, Krishna Prasad Kamisetty

**Affiliations:** 1 Pathology, Sri Devaraj Urs Medical College, Sri Devaraj Urs Academy of Higher Education and Research, Kolar, IND; 2 General Surgery, R L Jalappa Hospital &amp; Research Centre, Sri Devaraj Urs Academy of Higher Education and Research (SDUAHER), Kolar, IND

**Keywords:** laminin 332, lymphovascular invasion, perineural invasion, triple-negative breast carcinoma, infiltrating ductal carcinomas

## Abstract

Background: Breast carcinoma (BC) is the most common malignancy among women and is the leading cause of mortality among females. Triple-negative breast carcinoma (TNBC) is a diverse disease based on immunohistochemistry (IHC) and is estrogen receptor (ER) negative, progesterone receptor (PR) negative, and human epidermal growth factor (HER2) negative. TNBC has a distinct molecular profile, is more aggressive, lacks targeted therapies, and has a worse prognosis than other types of breast cancer. Laminin is a glycoprotein that plays several roles in cancer progression, including cell proliferation, invasion, metastasis, and epithelial-mesenchymal transition.

Aim and objectives: This study aimed to evaluate the immunohistochemical expression of laminin 332 in TNBCs and to study the association of laminin 332 expression with clinicopathological parameters of TNBCs.

Materials and methods: All the cases of TNBC received from the Department of Surgery at RL Jalappa Hospital and Research Institute to the Department of Pathology attached to Sri Devaraj Urs Medical College, Tamaka, Karnataka, from January 2019 to September 2024 were considered for the study. Both prospective and retrospective cases were considered. The data and paraffin blocks were retrieved from the archives of the Department of Pathology. Histopathological parameters of TNBC cases were studied, and laminin 332 IHC was performed. The association of IHC expression of laminin 332 and histopathological parameters was evaluated.

Results: Among 50 TNBC cases, 26 (56%) were elderly patients above 50 years of age. A higher proportion of cases, i.e., 23 (46%), were grade 3 tumors; 46 (92%) cases had infiltrating ductal carcinomas (IDC); 39 (78%) had lymphovascular invasion (LVI); 46 (92%) were without perineural invasion (PNI); and 22 (44%) had high-grade tumor-infiltrating lymphocytes (TILS). All the TNBC cases exhibited positivity for either a laminin 332 IHC score of 5 (64%) or a laminin 332 IHC score of 6 (36%). Laminin 332 IHC score of 5 (71.8%) was associated with the presence of LVI, and laminin IHC scores of 6* (*p-value 0.041) and 7 (63.6%) were associated with the absence of LVI, which has a statistically significant association with p-value 0.041.

Conclusions: All the TNBC patients were positive for laminin 322, but there was a statistically significant association only with lymphovascular invasion. TNBC, hence, exhibits aggressive behavior and is associated with unfavorable clinicopathological outcomes.

## Introduction

Breast carcinoma (BC) is the most prevalent malignancy among women, constituting 11.7% of all cancer cases. It represents the leading cause of mortality among females. The increasing prevalence of disease in both developed and developing countries presents a significant global threat [1].

In 2020, the worldwide incidence of breast cancer was 2,261,419 (11.7%), with 684,996 deaths (6.9%). Among females, the incidence rate of BC is the leading cause of cancer mortality worldwide [1]. In India, breast cancer has recently overtaken cervical carcinoma as the most common cancer among women, a change attributed to the gradual shift in lifestyle factors [1]. In India, the GLOBOCAN data for 2020 reveal that breast cancer is a significant health challenge, accounting for 13.5% (178361) of all cancer cases and 10.6% (90408) of cancer-related deaths. The cumulative risk stands at 2.81.1. Approximately one in four women were newly diagnosed and died due to BC in India [2]. The proportion of breast cancer in Bangalore is 34.4% [2]. Breast cancer exhibits a significant likelihood of recurrence and metastasis [3]. The reported prevalence of breast cancer in Kolar is 6.41% [3,4].

Triple-negative breast carcinoma (TNBC) is thought to behave more violently and has a worse prognosis than other forms of breast cancer. These cancers are distinguished by a lack of estrogen receptors (ER), progesterone receptors (PR), and HER2neu gene expression. A large number of these cases exhibit a basal-like appearance with laminin 332 overexpression [5]. Laminin is thought to be linked to the basal-like phenotype and BRCA1 deficiency [6].

Laminin is a heterotrimeric glycoprotein that performs a variety of functions both during embryonic development and in mature tissues [7]. During embryonic development, this extracellular matrix protein mediates cell attachment, migration, and tissue organization [8]. It also aids in cellular differentiation and survival, as well as the growth of embryonic stem cells [9]. Laminin constitutes a part of epithelial and vascular basement membranes within mature tissue, where it aids in the maintenance of cell adhesion and cohesion. Both epithelial and stromal cells secrete laminin, and it binds to integrin receptors on cell surfaces [9,10].

Laminin expression has been linked to carcinogenesis hallmarks such as cell proliferation, invasion, metastasis, and the epithelial-mesenchymal transition (EMT). Laminin 332 is located in the cytoplasm of tumor cells and at the interface between the tumor and the surrounding stroma. Basal cell carcinoma, advanced breast cancer, and prostatic cancer exhibit elevated laminin 332 expression levels [9].

TNBC exhibits a higher propensity for distant metastasis and recurrence post-treatment. Immunohistochemical studies indicate that approximately 70% of TNBCs exhibit positive expression of laminin 332 in BC [5].

Laminin 332 facilitates the migration of BC cells and is associated with tumor invasiveness [11]. Laminin 332 is widely recognized for its role in enhancing the motility of breast cancer cells via integrins and is linked to breast cancer metastasis [11,12].

This study aimed to evaluate the role of laminin 332 expression in TNBCs and study the association of laminin 332 expression with age, histological type, grade, and prognostic factors of TNBC.

## Materials and methods

The present study is a laboratory-based cross-sectional study conducted both prospectively (23 cases) and retrospectively (27 cases) from January 2019 to October 2024. Surgically resected lumpectomy and mastectomy specimens of TNBC received from the Department of Surgery at RL Jalappa Hospital and Research Institute and transferred to the Department of Pathology attached to Sri Devaraj Urs Medical College, Tamaka, Kolar, were considered for the study. Clinical details like age, body mass index (BMI), menopausal status, clinical presentation, site, and size of tumor were collected from case files. The data and paraffin blocks were retrieved from the archives of the Department of Pathology. Immunohistochemical staining for laminin 332 in histopathologically diagnosed cases of TNBC was performed.

Ethical clearance was obtained from the Central Ethics Committee of Sri Devaraj Urs Medical College, Tamaka, Kolar, with approval number SDUAHER/KLR/R&D Cell/06/2024-25. Prospective cases were included after obtaining informed consent, while retrospective cases were analyzed on the basis of anonymized data.

All cases of histopathologically and immunohistochemically confirmed TNBCs (ER, PR, HER2neu negative) with adequate tumor tissue and sufficient connective tissue stroma in paraffin blocks are included in the study, and cases that were previously subjected to surgery, chemotherapy, or radiotherapy were excluded from the study.

Methodology

The breast tissue fixed in 10% neutral buffered formalin and embedded in paraffin wax was considered for the study. Tissue sections were stained with hematoxylin and an eosin stain, and subsequently, immunostaining was done. The tissue sections were screened and analyzed for histomorphological features such as histopathological type, lymphovascular invasion, perineural invasion, grade, and stage of the tumor. ER, PR, Her2neu, and Ki67 expression of each case were noted from case files. Tissue sections were subjected to laminin 332 immunohistochemical staining.

Immunohistochemistry (IHC) staining procedure

Tissue sections were de-waxed and brought to distilled water, washed briefly for one to two minutes, and underwent antigen retrieval in a microwave oven for two cycles at 96 degrees Celsius for six minutes according to the standardization protocol for the antibody in citrate buffer pH 6.0/TRISEDTA pH 9, and then cooled for five to 10 minutes. These are washed in distilled water without letting the sections dry out and kept in 3% endogenous peroxidase for 10 minutes and washed in tris-buffered solution (TBS), pH 7.4, for two minutes. The sections are then subjected to primary antibodies (Diagnostic BioSystems code no. PDM 568, Pleasanton, CA) for 45 minutes to one hour, based on validation, at room temperature. The slides were washed twice with TBS for two minutes. Then the sections were subjected to secondary antibody (Diagnostic BioSystems code no. KP-5001) for 30 minutes, and slides were washed twice in TBS for two minutes. Sections were then subjected to diaminobenzidine tetrahydrochloride (DAB) chromogen for five minutes (R1-1 ml, R2-30 µL) and washed with distilled water. The sections were subjected to hematoxylin for 30 seconds and washed with TBS, followed by distilled water two times in two changes. The sections were dehydrated by three changes of absolute alcohol, cleared with two changes of xylene for two minutes, and mounted with distyrene, plasticizer, and xylene (DPX). Skin was taken as control.

Interpretation of staining

**Figure 1:**
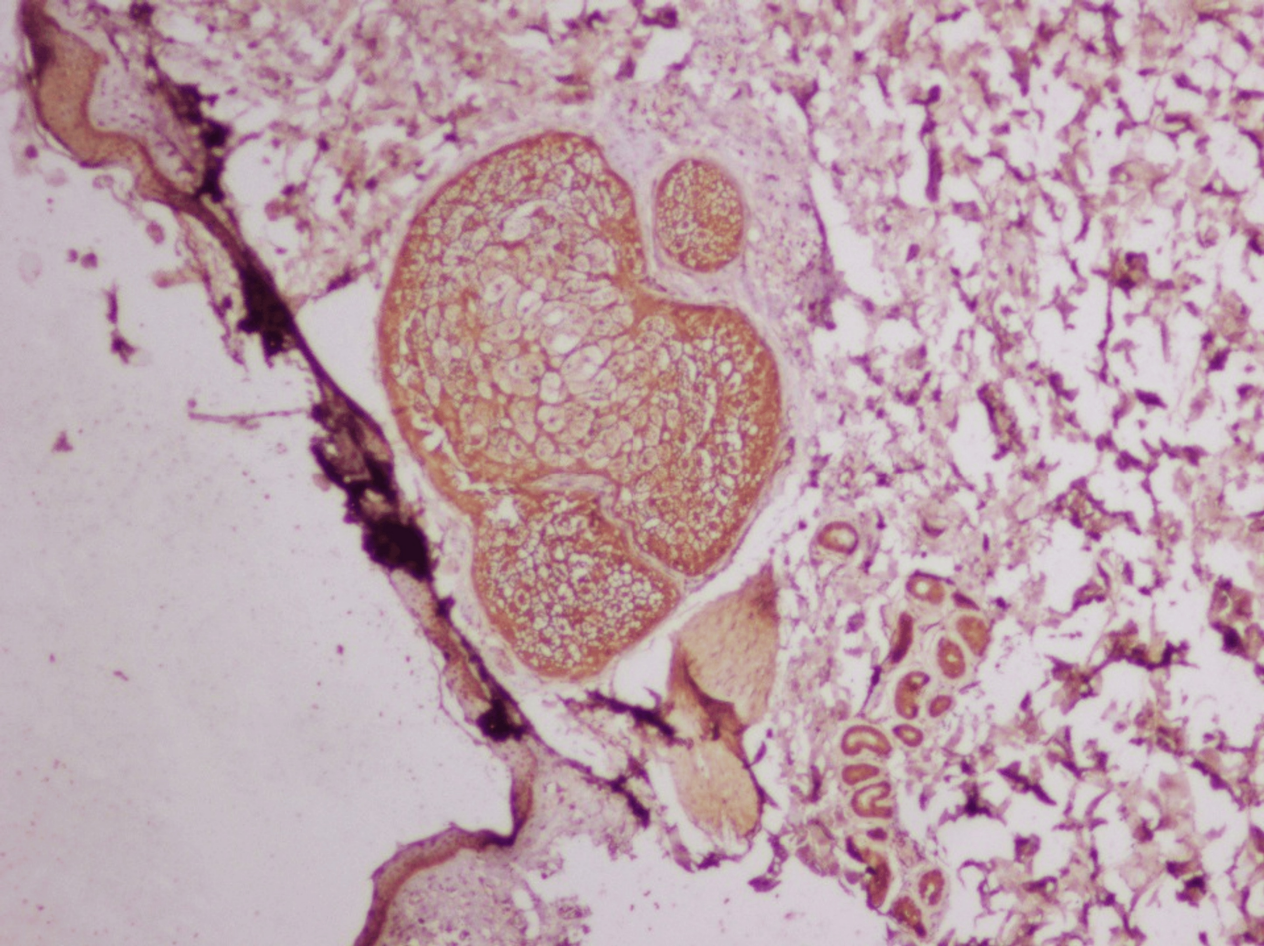
Microphotograph of skin used as positive control (laminin 332, 200X)

**Figure 2:**
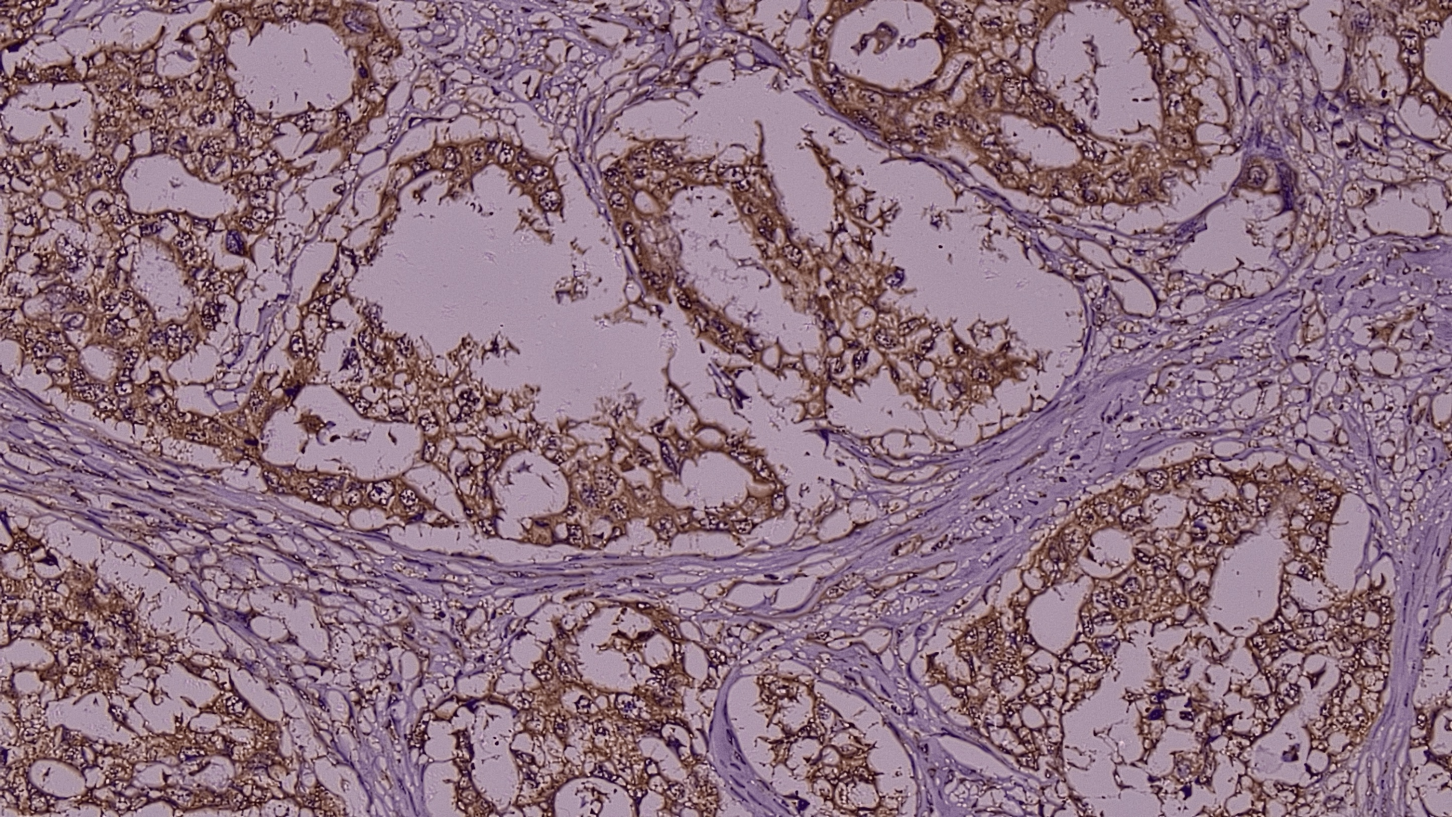
Microphotograph showing weak intensity (light yellow)/low proportion of laminin immunohistochemistry (IHC) staining (laminin 332 IHC, 400X)

**Figure 3:**
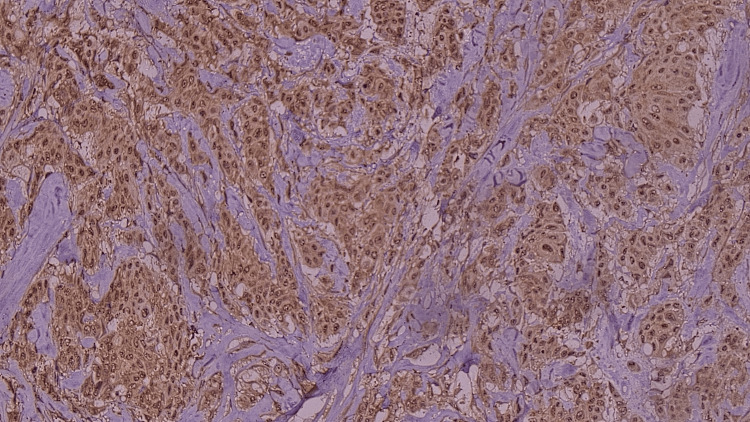
Microphotograph showing moderate intensity (yellow/brown)/intermediate proportion of laminin immunohistochemistry (IHC) staining (laminin 332 IHC, 400X)

**Figure 4:**
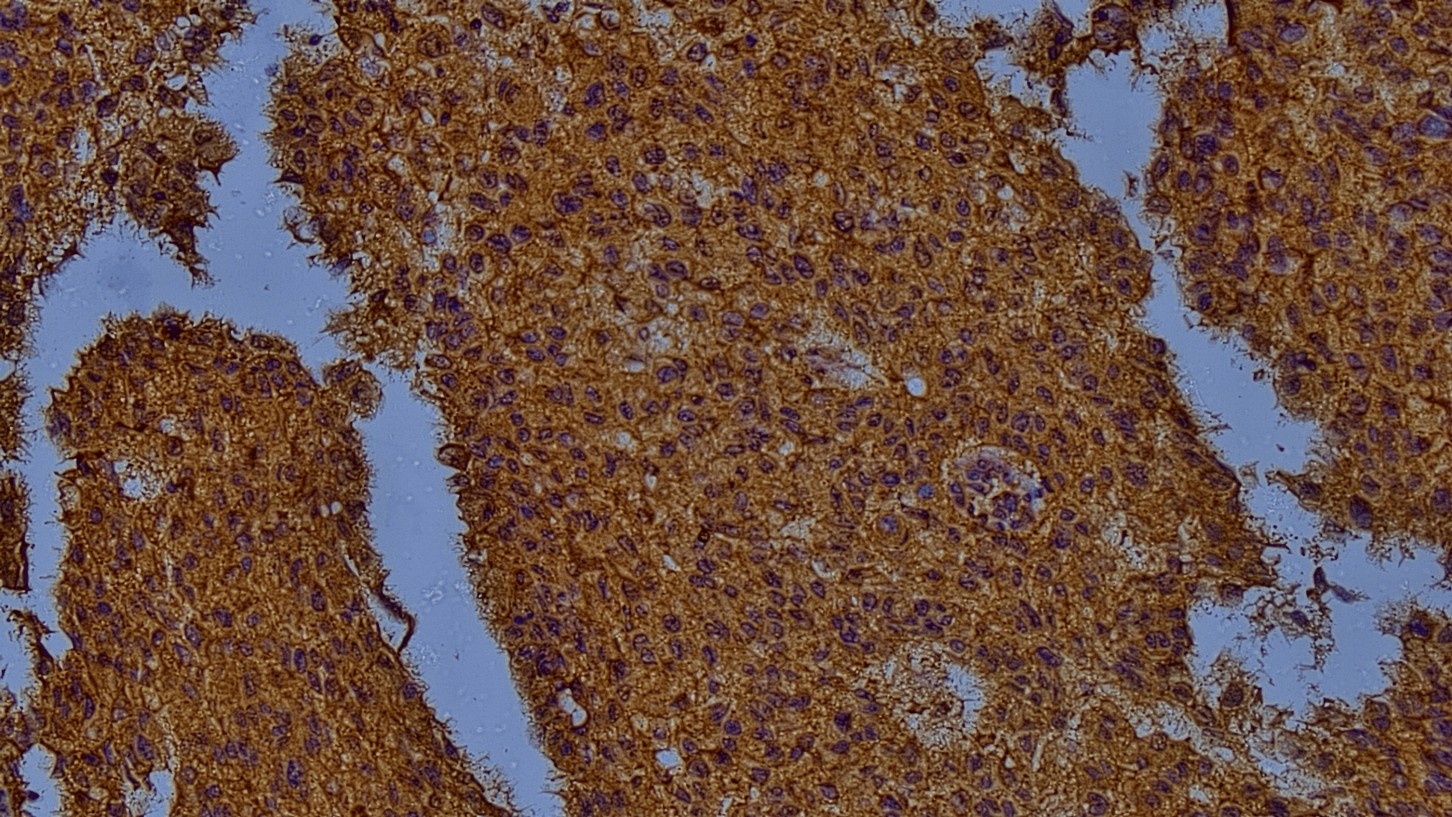
Microphotograph showing strong intensity(brown)/high proportion of laminin immunohistochemistry (IHC) staining (laminin 332 IHC, 400X)

Skin was used as a positive control (Figure 1), and breast tissue without primary antibodies was used as a negative control. The cytoplasmic stain in 10 consecutive representative fields was examined at x10X and x40X. Intensity of staining and proportionate score of staining were evaluated. The laminin expression was measured using a semiquantitative four-tier intensity score (Table 1 and Figures 2-4)

**Table 1:** The laminin 332 expression was measured using a semiquantitative four-tier system of intensity score Source: [5]

Grade	Immunoreactivity Intensity Score	Interpretation
I	0,1+	Absent, weak staining (light yellow)
II	2+	Intermediate or Moderate staining (yellow/brown)
III	3+	strong staining (brown)

Interpretation of the proportionate score for laminin 332 IHC was done (Table 2) [12,13,14].

**Table 2:** Interpretation of proportionate score for laminin 332 immunohistochemistry Source: [12-14]

Percentage of positive cells	Proportionate score
<1%	0
1-5%	1
5-30%	2
>30%	3

Both the laminin 332 intensity IHC score and the proportionate score expressed by tumour cells were added, and the final grade was obtained (Table 3).

**Table 3:** Calculation of final grade for laminin 332 immunohistochemistry score

Final grade	Intensity score + Proportionate score
Grade 1	1-3
Grade 2	4-6
Grade 3	7-9

Statistical analysis

Data was entered into a Microsoft Excel data sheet (Microsoft Corp., Redmond, WA, USA) and was analyzed using IBM SPSS Statistics software, version 22 (IBM Corp., Armonk, NY, USA). Categorical data were represented in the form of frequencies and proportions. The chi-square test or Fisher’s exact test (for 2x2 tables only) was used as a test of significance for qualitative data. Continuous data were represented as mean and standard deviation (SD). An independent t-test was used as a test of significance to identify the mean difference between two quantitative variables. MS Excel and MS Word (Microsoft Corp.) were used to obtain various types of graphs. A p-value of <0.05 was considered statistically significant after assuming all the rules of statistical tests.

## Results

In the present study, clinical and histopathological parameters like age, parity, menopausal status, BMI, tumor size, pathological staging, histopathological diagnosis, lymphovascular invasion, perineural invasion, Nottingham Prognostic Index, modified Scarff-Bloom-Richardson grading, tumor-infiltrating lymphocytes, and their association with laminin 332 IHC were studied in 50 TNBC patients.

The present study included 50 participants, of whom 22 (44.0%) were ≤ 50 years of age and 28 (56.0%) were ≥ 50 years, with a mean of 50.3 years and a median of 54.5 years (Table 4).

**Table 4:** Clinicopathological parameters pTNM: pathological tumor-node-metastasis

Parameters	Frequency	Percent
Age group		
<50 years	22	44.0
>50 years	28	56.0
Parity		
P1	3	6.0
P2	26	52.0
P3 and above	21	42.0
Menopausal status		
Post menopausal	29	58.0
Premenopausal	21	42.0
Body mass index		
Underweight	4	8
Normal	42	84
Overweight	4	8
Laterality		
Left	25	50.0
Right	25	50.0
Tumor size		
<2 cm	5	10.0
2-5 cm	28	56.0
>5 cm	17	34.0
pT stage		
pT1	6	12.0
pT2	27	54.0
pT3	10	20.0
pT4	7	14.0
pN stage		
N0	39	78.0
N1	9	18.0
N3	2	4.0
pTNM stage		
Stage 1	5	10.0
Stage 2	37	54.0
Stage 3	8	16.0
Histopathological diagnosis		
IDC	46	32.0
IDC with medullary differentiation	2	24.0
Poorly differentiated infiltrating ductal carcinomas (IDC)	2	24.0
Lymphovascular invasion		
Absent	11	22.0
Present	39	78.0
Perineural invasion		
Absent	46	92
Present	4	8
Tumor infiltrating infiltrates		
Low grade	16	32.0
Intermediate grade	12	24.0
High grade	22	44.0
Modified Scarff-Bloom-Richardson grading		
Grade 1	6	12
Grade 2	21	42
Grade 3	23	46
Nottingham Prognostic Index		
2-2.4	0	0
2.4-3.4	22	44
3.4-5.4	28	46
Laminin 332 immunohistochemistry (IHC) scoring		
5	32	64.0
6	18	36.0

In the present study, laminin 332 IHC expression was seen in all study populations. Out of this, 32 (64%) of the study population have a laminin 332 IHC score of 5, and 18 (36%) have an IHC score of 5. The majority of the study population had a laminin 332 IHC score of 5 (Table 4).

Fifteen out of 22 (68.2%) of the study population expressed a laminin IHC score of 5, and seven out of 22 (31.8%) expressed a laminin IHC score of 6in the <50 years age group, and 17 out of 28 (60.7%) of the study population expressed a laminin IHC score of 5, and 11 out of 28 (39.3%) expressed a laminin IHC score of 6 in the >55 years age group, with no statistical association with a p-value of 0.803 (Table 5).

**Table 5:** Correlation of clinicopathological parameters with laminin 332 immunohistochemistry(IHC) Chi-square value of age: 0.298; menopausal status: 0.112; parity: 0.747; tumor size: 0.413; Modified Scarff-Bloom-Richardson grade: 1.596; laterality: 0.347; pT stage: 1,616; pN stage: 2.739; pTNM stage: 4.933; lymphovascular invasion: 4.675; perineural invasion: 0.370 pTNM: pathological tumor-node-metastasis

Parameters	IHC 5		IHC 6		P-value
	N	%	N	%	
Age					
<50 years	15	68.2%	7	31.8%	0.803
>50 years	17	60.7%	11	39.3%	
Menopausal status					
Post menopausal	18	62.1%	11	37.9%	0.774
Premenopausal	14	66.7%	7	33.3%	
Parity					
P1	2	66.7%	1	33.3%	0.688
P2	18	69.2%	8	30.8%	
P3 and above	12	57.1%	9	42.9%	
Tumour size					
<2 cm	3	60.0%	2	40.0%	0.813
2-5 cm	19	67.9%	9	32.1%	
>5 cm	10	58.8%	7	41.2%	
Modified Scarff-Bloom-Richardson grade					
Grade 1	5	83.3%	1	16.7%	0.450
Grade 2	14	66.7%	7	33.3%	
Grade 3	13	56.5%	10	43.5%	
Laterality					
Left	17	68.0%	8	32.0%	0.769
Right	15	60.0%	10	40.0%	
pT stage					
pT1	4	66.7%	2	33.3%	0.656
pT2	18	66.7%	9	33.3%	
pT3	7	70.0%	3	30.0%	
pT4	3	42.9%	4	57.1%	
pN stage					
N0	26	66.7%	13	33.3%	0.254
N1	4	44.4%	5	55.6%	
N3	2	100.0%	0	0.0%	
pTNM stage					
Stage 1	4	80%	1	20%	0.294
Stage 2	24	64.8%	13	35.2%	
Stage 3	4	50%	4	50%	
Lymphovascular invasion					
Absent	4	36.4%	7	63.6%	0.041
Present	28	71.8%	11	28.2%	
Perineural invasion					
Absent	30	65.2%	16	34.8%	0.612
Present	2	50.0%	2	50.0%	

## Discussion

Breast cancer is the most common malignancy worldwide. In India, the age-adjusted incidence of BC among females is 25.8 per 100,000, and the death rate is 12.7 per 100,000 [4]. Molecular subtypes of breast carcinoma are luminal A-like, luminal B-like (HER2-negative), luminal B-like (HER2-positive), HER2-enriched, and basal-like. TNBCs, especially the basal-like type, are aggressive [15]. Laminin, a diagnostic molecule, serves as a prognostic marker. Its staining pattern changes from regular and linear in non-neoplastic breast tissue to irregular and disrupted in carcinomas [5].

In IDC, laminin staining reveals basement membrane material around tumor cell groups, suggesting that tumor cells produce laminin. This indicates variable ability to produce basement membrane components, with stroma playing a role in synthesis. Laminin 332 is highly expressed in epithelial tumors, accumulating at the tumor-stroma interface [11].

BCs exhibit heterogeneous laminin distribution, with production limited to tumor cells adjacent to stroma, indicating the stroma's role in basement membrane synthesis. This pattern varies across histologic types and differentiation degrees [11].

In the present study, a total of 50 TNBC cases were studied. The association between histopathological parameters and laminin 332 immunostaining was studied. Among the study group, 100% of the study population expressed laminin 332, which is not in concordance with studies done by Rath et al. [5]. In the current study 56% of the study population were ≥ 50 years of age, and 44% were ≤ 50 years of age and identified to be positive for laminin 332. The study by Rath et al. [5] documented that 89.29% were over 50 years old, but only 56% of this group were positive for laminin 332.

In our study, the majority of patients had a tumor size of 2-5 cm, with 28 (56%) with a score of 5 (67.9%) and a score of 6 (32.1%) positivity, which was not in concordance with the study conducted by Rath et al. [5], where the majority had a tumor size of >5 cm, with 30 (53.57%) where 86.67% were laminin 332 negative and 13.33% were laminin 332 positive.

In the present study, the majority of patients had Grade 3 disease, 23 (46%), with laminin 332 scores of five (56.5%) and 6 (43.5%) positivity, which was not in concordance with the study conducted by Rath et al. [5], where the majority had Grade 3 disease (64.29%), among which 66.7% did not show laminin 332 positivity.

In the present study, the majority of patients, nine (78%), had lymphovascular invasion, among whom 71.8% had score-5 and 28.2% had score-6 positivity, which was not in concordance with the study conducted by Rath et al. [5], where the majority (64.29%) had lymphovascular invasion, among whom only 22.22% documented laminin 332 positivity.

There were limited studies to compare the association of laminin 332 with perineural invasion, tumor-infiltrating lymphocytes, the Nottingham Prognostic Index, and pathological tumor-node-metastasis (pTNM).

Limitations of the study were a small sample size and data collected from a single hospital; a multicenter study is needed to assess the status in a better way.

## Conclusions

TNBCs exhibit aggressive behavior and are associated with unfavorable clinicopathological outcomes. Laminin immunostaining may serve as a prospective prognostic marker for predicting outcomes in patients with TNBC. Utilizing laminin antibodies as a potent chemotherapeutic drug can facilitate effective cancer management and enhance patient survival. In this study, we documented that all the TNBC patients showed expression for laminin 322, but there was a statistically significant association only with lymphovascular invasion.
